# Bio-derived three-dimensional hierarchical carbon-graphene-TiO_2_ as electrode for supercapacitors

**DOI:** 10.1038/s41598-018-22742-7

**Published:** 2018-03-13

**Authors:** Lili Jiang, Zhifeng Ren, Shuo Chen, Qinyong Zhang, Xiong Lu, Hongping Zhang, Guojiang Wan

**Affiliations:** 1 Key Laboratory of Fluid and Power Machinery of Ministry of Education, Center for Advanced Materials and Energy, School of Materials Science and Engineering, Xihua University, Chengdu, 610039 China; 2 Department of Physics and TcSUH, University of Houston, 3201 Cullen Blvd, Houston, Texas 77204 USA; 3 Key Lab of Advanced Technologies of Materials, Ministry of Education, School of Materials Science and Engineering, Southwest Jiaotong University, Chengdu, 610031 Sichuan China; 4 School of Materials Science and Engineering, Southwest University of Science and Technology, Mianyang, 621010 Sichuan China

## Abstract

This paper reports a novel loofah-derived hierarchical scaffold to obtain three-dimensional biocarbon-graphene-TiO_2_ (BC-G-TiO_2_) composite materials as electrodes for supercapacitors. The loofah scaffold was first loaded with G and TiO_2_ by immersing, squeezing, and loosening into the mixed solution of graphene oxide and titania, and then carbonized at 900 **°**C to form the BC-G-TiO_2_ composite. The synergistic effects of the naturally hierarchical biocarbon structure, graphene, and TiO_2_ nanoparticles on the electrochemical properties are analyzed. The biocarbon provides a high interconnection and an easy accessibility surface for the electrolyte. Graphene bridged the BC and TiO_2_ nanoparticles, improved the conductivity of the BC-G-TiO_2_ composite, and increased the electron transfer efficiency. TiO_2_ nanoparticles also contributed to the pesudocapacitance and electrochemical stability.

## Introduction

Supercapacitors are energy storage devices and play a significant role in utilization of clean energy^1^. According to one of the storage mechanisms of supercapacitors, electrochemical double-layer capacitors (EDLCs) physically store energy by adsorption of ions at the electrode/electrolyte interfaces. Thus, a high specific surface area for the electrodes improves the gravimetric specific capacitance of a supercapacitor. However, a high surface area decreases the volumetric capacitance because of the high porosity^2^. Carbon-based materials with porous structures have been widely studied as the electrode materials for supercapacitors because of their high specific surface area, as well as their superior electrochemical stability. In these researches, biomass was firstly carbonized and crushed into carbon powder, which was subsequently stacked to construct porous carbon structure. For example, porous carbon has been prepared using carbonization and activation of carbon precursor particles like those from fruit peels^3^, bamboo^4^, and seaweed^5^. Though the above bio-inspired porous carbon may have high specific surface area, this discontinuous and uninterconnected porous structure makes inefficient use of specific surface area owing to the losing of ion-accessible surface for the electrolyte. Moreover, the chemical activation process which is carried to increase the specific surface area or hydrophilicity is not eco-friendly by using harmful chemicals. In addition, according to Langlois’ semi-empirical formula $\rho \approx K\bullet \frac{4}{1-\theta }\bullet {\rho }_{0}$, where $\rho$ is the resistivity of porous materials, ${\rho }_{0}$ the resistivity of dense materials, $\theta \,\,$the porosity, and *K* the correction constant^6^, a higher porosity leads to a lower conductivity. However, high electrical conductivity is very important for fast charging and discharging of supercapacitors^7^. Hence, a compromise between the specific area and porosity needs to be reached for not only high capacity but also high electrical conductivity.

Recently, the utilization of natural structure of biomass attracted a tremendous attention because of its continuity, interconnection, and hierarchy of the structure. Liu *et al*. obtained a honeycomb-like macroporous carbon from mollusk shell through a low cost and eco-friendly fabrication process^8^. This porous carbon inherits the naturally highly interconnected hexangular channels of the mollusk shell and exhibits excellent electronic conductivity, which ensures fast electron transfer and effective electrolyte penetration, making it a good candidate as a supercapacitor electrode. After incorporating Co_3_O_4_ onto the porous carbon structure, the composite exhibited an outstanding electrochemical performance (a high specific capacitance of 1307 F g^−1^ at 1 A g^−1^). Wu *et al*. prepared a sponge-like carbonaceous hydrogels and aerogels using watermelon as the carbon source^9^. These porous carbonaceous gels are good candidates as scaffolds for synthesis of three-dimensional composite materials.

However, according to another storage mechanism of supercapacitors, pseudocapacitors which store charges through rapid and reversible surface or near-surface Faradic reactions, leading to much higher specific capacitance than EDLCs^10–13^. Metal oxides, such as MnO_2_^14–16^, RuO_2_^17,18^, CeO_2_^19,20^, and TiO_2_^21–23^ are promising electrode materials for supercapacitors because of their excellent pseudocapacitance performance. Even though TiO_2_ nanoparticles have been widely studied as an electroactive material as supercapacitor electrodes because of their good chemical stability, electrochemical activity, low cost, low toxicity, and environmental friendliness, the intrinsically low conductivity significantly decreases its capacitance performance. Fortunately, the electrical conductivity can be effectively improved by incorporating highly conductive carbon-supporting materials^24,25^, including, for example, graphene that has high electrical conductivity and high surface area^26–28^. But unfortunately the electrochemical performance of graphene-based materials is strongly affected by the aggregation or restacking that inevitably occurs as a result of inter-sheet van der Waals attractions^29,30^. Yang *et al*. inspired by biomass structures, exploited water molecules to prevent the restacking of graphene sheets and obtained graphene films with an interconnected structure^31^. The results showed that a biomass structure could be applied in combination with graphene or as a substrate for a graphene-based composite to prevent aggregation and restacking, further improve the ion-accessible surface area, and expose the abundant active sites to electrolyte ions.

In this study, a completely natural highly interconnected and interpenetrated loofah-derived hierarchical structure was studied as a scaffold for graphene and TiO_2_ composite, which inherits the natural highly interconnected and interpenetrated porous structure and abundance ion-accessible surfaces for electrolyte. A three-dimensional carbon-graphene-TiO_2_ composite electrode was studied as a supercapacitor electrode using a convenient and eco-friendly one-pot process and high-temperature treatment. The electrochemical performances of carbon, carbon-graphene, and carbon-graphene-TiO_2_ composites were comparatively studied.

## Results

**Figure 1:**
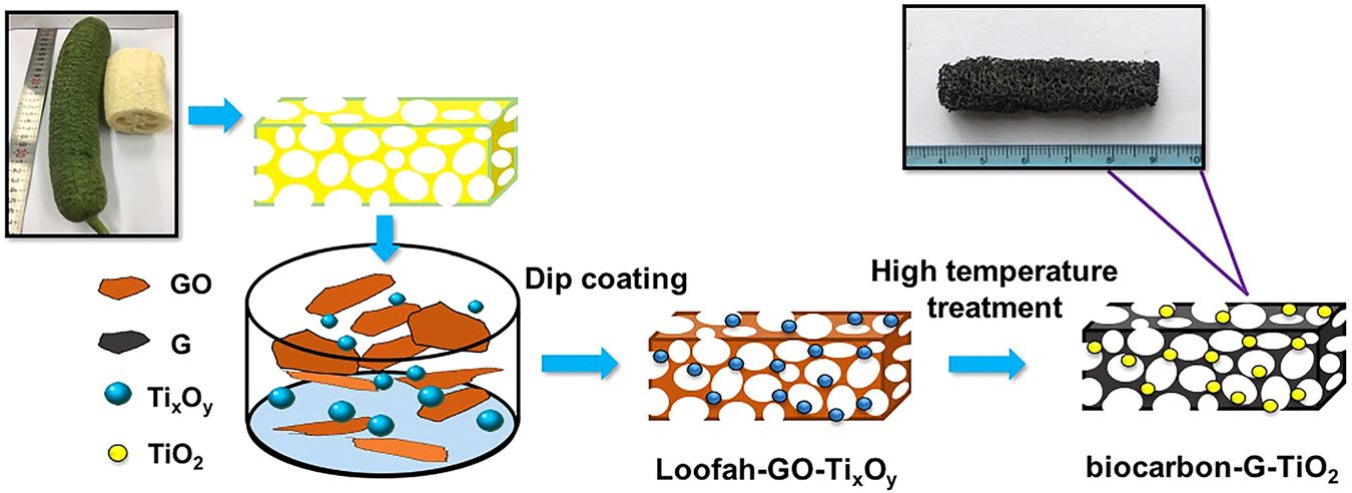
Illustration and details of BC-G-TiO_2_ preparation process.

**Figure 2:**
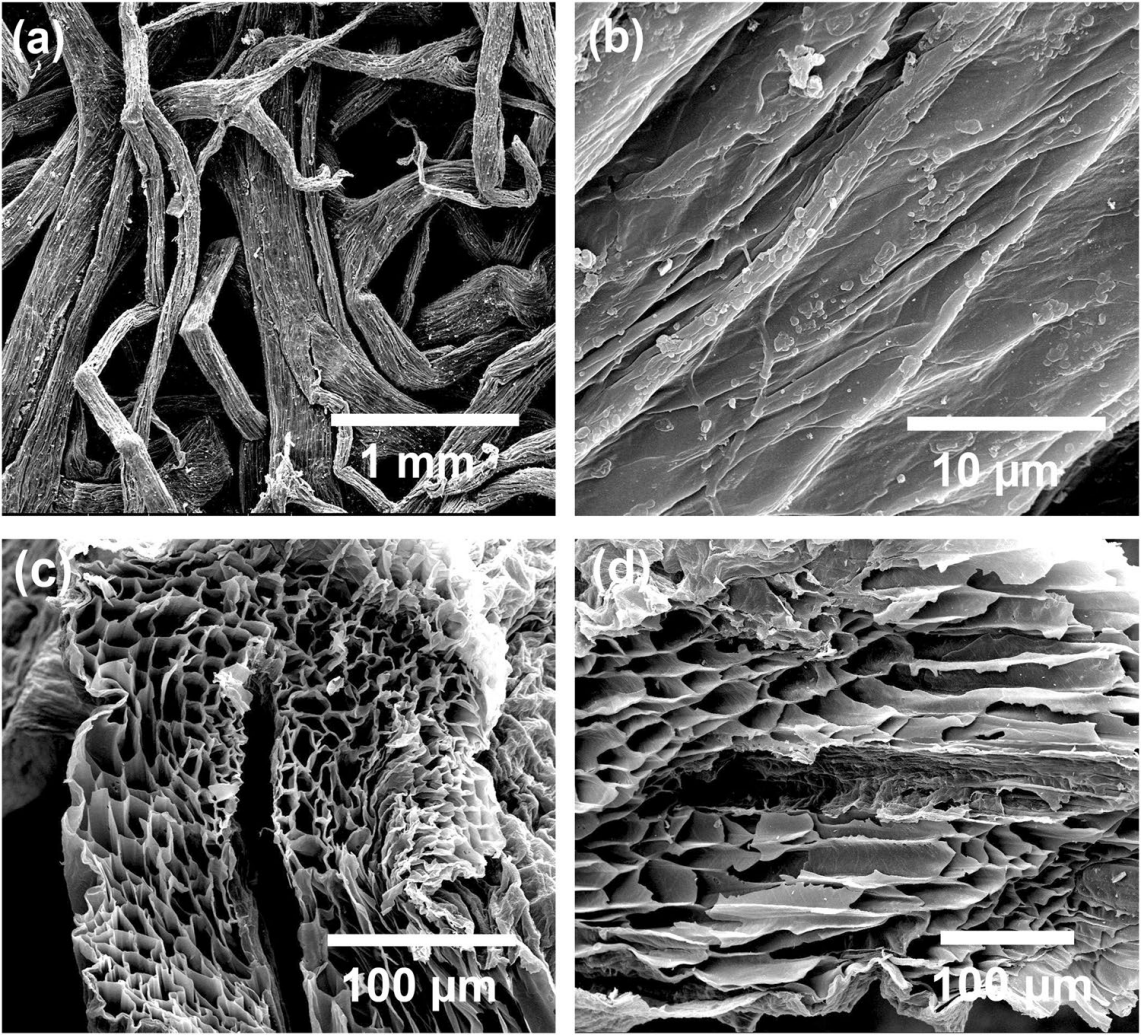
SEM micrographs of carbonized loofah fiber: (**a**) porous structure of carbonized loofah fiber, (**b**) surface morphology of carbonized loofah fiber, (**c**) cross-sectional view of carbonized loofah fiber, and (**d**) oblique section view of carbonized loofah fiber.

A graph of the as-prepared BC-G-TiO_2_ composite is shown in Fig. 1. It proves that the BC-G-TiO_2_ composite retains the biological structure of the loofah. SEM micrographs reveal the morphology of the BC after the high-temperature treatment (Fig. 2). The irregular carbonized loofah fibers and interconnected structure inherites from the natural structure of the loofah are shown in Fig. 2a. Figure 2b shows the surface morphology and naturally wrinkled surface of the carbonized loofah fibers. The cross-sectional view and oblique view of the carbonized loofah fibers are displayed in Fig. 2c, d, respectively, which reveal the inner microscale tubular structure within a single carbonized loofah fiber. This indicates that, after carbonization, the biological fiber network structure and inner porous structure of the loofah are well retained, which proves the high interconnection and hierarchy of BC. Moreover, the average diameter of the inner microscale tubes is approximately 1.3 µm, and the total surface area of the BC (1 × 1 × 1 cm^3^) is ~3.8 × 10^4^ m^2^ g^−1^.

**Figure 3:**
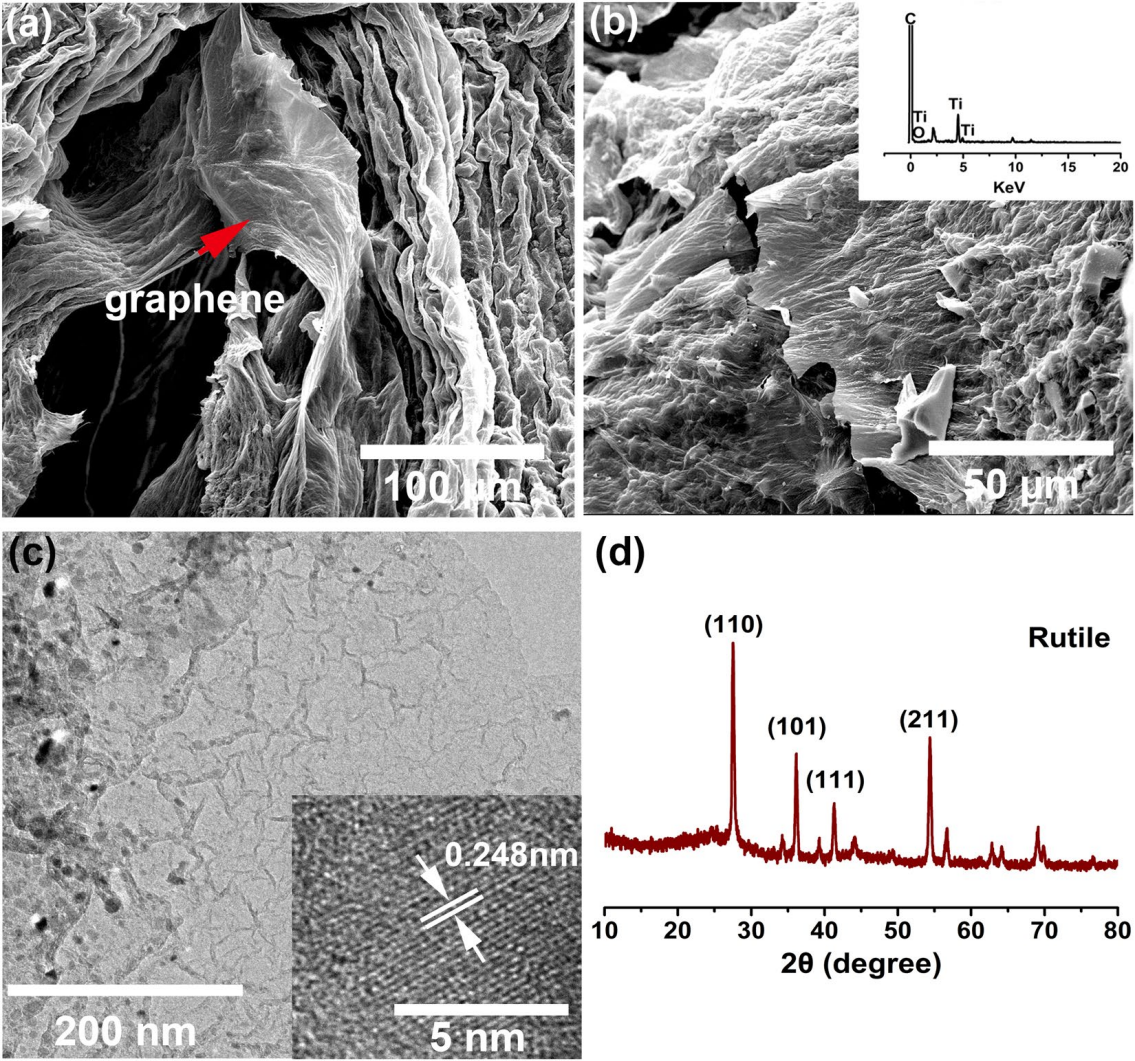
SEM micrographs of BC-G and BC-G-TiO_2_: (**a**) BC-G, (**b**) BC-G-TiO_2_, the EDX spectra of BC-G-TiO_2_ as inset, (**c**) HETEM images of BC-G-TiO_2_, and (**d**) XRD spectrum of BC-G-TiO_2_ obtained at 900 °C.

The morphologies of the BC-G and BC-G-TiO_2_ are shown in Fig. 3. The silk-like graphene sheets coat the carbonized loofah fiber surface (Fig. 3a). Figure 3b shows the morphology of the BC-G-TiO_2_ composite. The TiO_2_ nanoparticles are distributed and covered by graphene sheets. The EDX spectrum of the BC-G-TiO_2_ composite proves the existence of titania oxide (inset in Fig. 3b). Figure 3c shows the high-resolution TEM image of BC-G-TiO_2_ composite, which proves the typical sheets and wrinkle structure of graphene, and the TiO_2_ nanoparticles are evenly distributed or covered by graphene sheets. The width of the lattice fringes was 0.248 nm, as observed in Fig. 3c corresponding to the (101) plane of rutile. The further proof of the evenly distribution of TiO_2_ nanoparticles is provide in an EDX mapping spectrum (Figure S1). The XRD spectrum of BC-G-TiO_2_ in Fig. 3d shows the existence the rutile phase of TiO_2_. The characteristic peaks at 27.43°, 36.08°, 41.24°, and 54.32° correspond to the (110), (101), (111), and (211) crystallographic planes of rutile, respectively (JCPDS No. 01-078-2485). It is recognized that the dome peaks between 23.00° to 26.00° correspond to graphene in some previous studies^32,33^.

**Figure 4:**
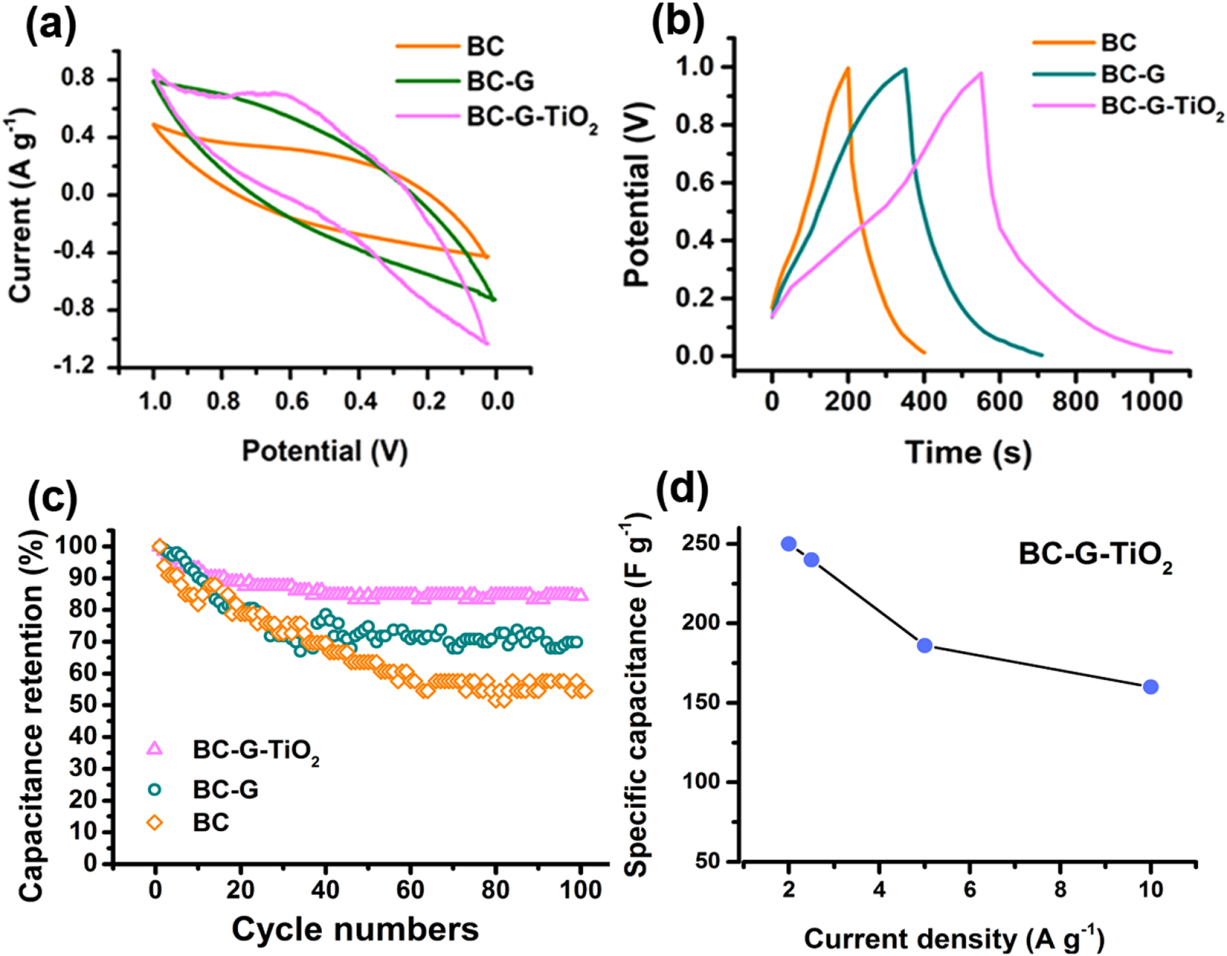
Electrochemical performances of BC, BC-G, and BC-G-TiO_2_ composite electrodes: (**a**) CV curves at scan rate of 10 mV s^−1^, (**b**) galvanostatic charge and discharge curves, (**c**) cycle stability performance at current of 2 A g^−1^, and (**d**) specific capacitances calculated from discharge curves at different current densities.

The electrochemical performances of BC-based three-dimensional electrodes were investigated using CV, galvanostatic charge-discharge, cycle stability performance, and rate performance characterizations. As shown in Fig. 4a, the shape of the CV curve of the BC electrode indicates that its capacitance mainly comes from EDLC. The addition of G increases not only the integral area of the hysteresis loop, but also the current density of the BC-G electrode. This indicates that the incorporation of G improves the capacitance of the BC-G electrode, which also mainly comes from EDLC. After the addition of TiO_2_ nanoparticles, the integral area of the hysteresis loop is significantly increased. The larger integral area indicates that the higher capacitance of the BC-G-TiO_2_ electrode is a consequence of the incorporation of TiO_2_ nanoparticles. It is observed that the additional pseudocapacitance of the BC-G-TiO_2_ comes from the reversible redox reactions of TiO_2_, which occur at ~0.6 V and ~0.3 V on the CV curve of BC-G-TiO_2_. Thus, the capacitance of the BC-G-TiO_2_ electrode depends on both the EDLC and pseudocapacitance.

The galvanostatic charge-discharge curves also indicate that the BC-G-TiO_2_ has the longest discharging time (Fig. 4b). The capacitance values were calculated from the galvanostatic charge-discharge curves at a current density of 2 A g^−1^. The BC electrode achieves a capacitance of 100.2 F g^−1^, which is lower than that of the BC-G (179.5 F g^−1^). After the addition of TiO_2_ nanoparticles, the BC-G-TiO_2_ sample electrode achieves the highest capacitance of 250.8 F g^−1^ among all these prepared samples.

The cycle stability performance of the BC-based electrode is displayed in Fig. 4c. The best stability is obtained from the BC-G-TiO_2_, with 84.4% of the capacitance retained after 100 charge-discharge cycles. In contrast, the BC and BC-G electrodes have lower stabilities than the BC-G-TiO_2_, with 54.5% and 69.9% of the capacitance retained, respectively. Figure 4d presents the rate performance of sample BC-G-TiO_2_. It indicates that the specific capacitance of the BC-G-TiO_2_ decreases with an increase in the current density. The capacity retention of 64.1% at 10 A g^−1^ proves the good rate performance of this three-dimensional hierarchical porous electrode.

**Figure 5:**
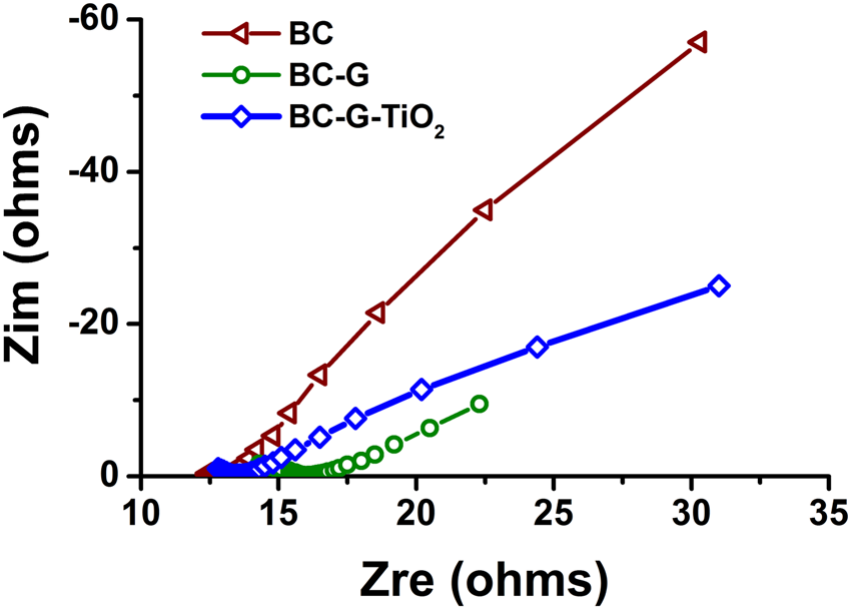
Nyquist plots for the samples at a direct current bias of 0 V with a sinusoidal signal of 20 mV over the frequency range of 200 kHz to 0.01 Hz.

Electrochemical impedance spectroscopy (EIS) was used to gain a fundamental understanding of the effects of graphene and TiO_2_ on the conductivity of the three-dimensional BC-based samples. Figure 5 shows the typical Nyquist plots of the BC-based samples. These curves demonstrate that the additions of G and TiO_2_ have different effects on the resistivity of the BC-based samples. The resistivity of the BC-G is significantly decreased by incorporation of graphene, whereas the addition of TiO_2_ nanoparticles increased the resistivity of the BC-G-TiO_2_ sample because of the semiconductor property of TiO_2_.

## Discussion

**Figure 6:**
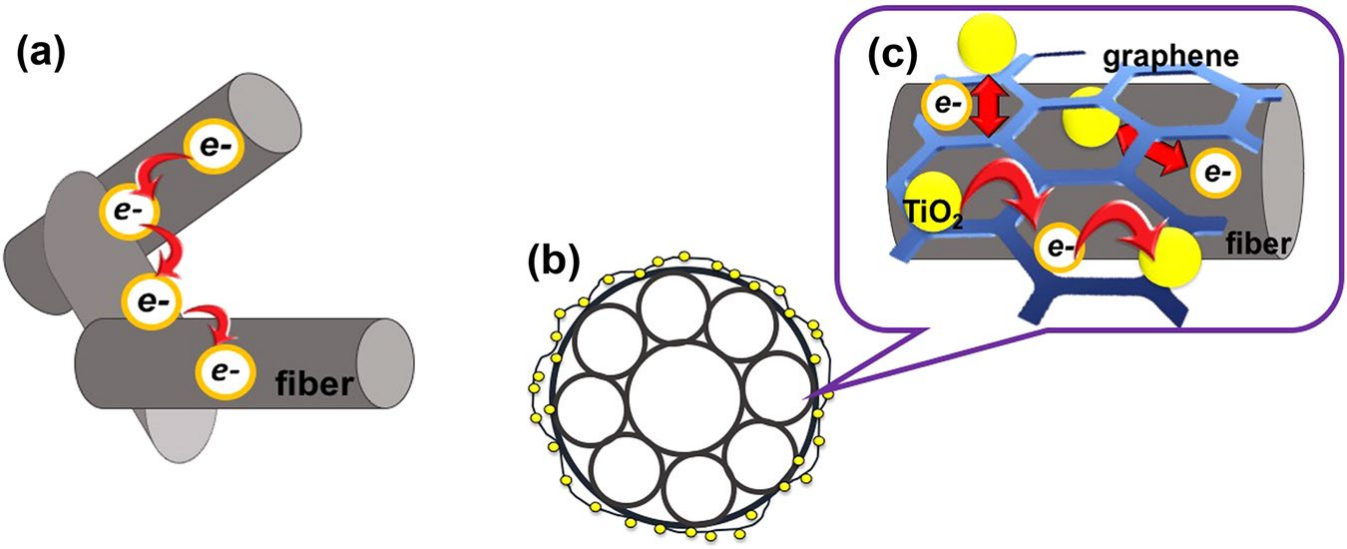
(**a**) Illustration of electron transfer in BC, (**b**) illustration of cross-section of one fiber of BC-G-TiO_2_ composite, and (**c**) illustration of electron transfer among fibers, TiO_2_, and graphene.

In this study, a completely natural highly interconnected and interpenetrated loofah-derived hierarchical structure design for three-dimensional BC-G-TiO_2_ composite electrode materials for supercapacitors was successfully obtained and exhibited high electrochemical performance properties. This high electrochemical performance was ascribed to the synergistic effects of all three phases of the BC-G-TiO_2_. The significant effects of BC on the electrochemical performance of the BC-G-TiO_2_ included two aspects: (i) the BC scaffold, which retained the natural hierarchy and interconnection of the loofah, facilitated the electron transfer in the porous structure (Figs 2a and 6a). On one hand, during the electrochemical process, the carbonized loofah fibers provided a pathway for electron-transport, just like wires, which ensured the efficient transfer of electrons within the carbonized loofah fiber^4,34,35^. On the other hand, the highly interconnected carbonized loofah fibers allowed a facile electron transfer throughout the entire porous structure of the BC because of the relatively shorter diffusion distance and lower ion-transport resistance^36,37^. (ii) The BC scaffold increased the ion-accessible surface area for the electrolyte. Figure 2 shows the internal microtubes of the carbonized loofah fiber. This inner microtube structure of the loofah transported H_2_O molecules and ions and exchange energy to keep alive. Hence, after the carbonization process, these microtubes within the fiber not only minimized the diffusion distances to the interior surfaces but also provided a more ion-accessible surface to strengthen the EDLC behavior. Similar macro-dimensional effects on the EDLC behavior with a porous texture have already been demonstrated^38–41^.

The roles of the graphene in the composite electrodes include three aspects: (i) graphene contributes to the improvement of capacitance because of its high specific surface area and intrinsic capacitance^29^. (ii) Graphene improves the conductivity of the samples, demonstrated in Fig. 5 by the EIS results. Additionally, graphene links the BC and TiO_2_ nanoparticles. The conductive mechanism of carbon is based on its sp^2^-hybridized structure, which is similar to graphene. Hence, there is no block when electrons transfer between the BC and graphene. The TiO_2_ nanoparticles distributed on the surface of the BC, encapsulated and connected by graphene (as shown in Fig. 3b,c), allow a highly efficient electron transfer among the three phases of the composite. (iii) Graphene provides more reactive sites for the TiO_2_, providing an efficient pathway for electron transfer among the TiO_2_ nanoparticles (as shown in Fig. 6b, c). Some similar conclusions have also been well documented, for example, Ramadoss *et al*.^42^ used a simple and fast microwave-assisted process to obtain a graphene-TiO_2_ hybrid and proved that it allowed the easy occurrence of electron transfer between the graphene and TiO_2_. Chen *et al*.^43^ also proved that the electron can easily transfer among graphene and TiO_2_ hybrid using First-principle calculations.

TiO_2_ nanoparticles have significant effects on the improvement of the electrochemical performance of BC-G-TiO_2_ because of its pseudocapacitance behavior. The charge storage mechanism of TiO_2_ has been discussed in our previous studies^44^. According to the following equation:

1 $${(Ti{O}_{2})}_{surface}+{H}_{3}{O}^{+}\leftrightarrow {(Ti{O}_{2}^{-}{H}_{3}{O}^{+})}_{surface}$$

during the electrochemical process, when the cation intercalation and deintercalation reaction occurs, the *H*_3_*O*^+^ in the aqueous electrolyte is adsorbed on the surface of the TiO_2_ nanoparticles, and the charge storage process takes place. In contrast, the loss of charge completes the subsequent deintercalation reaction, and the discharge process happens.

The electrochemical stability study suggested that both the graphene and TiO_2_ affected the electrochemical stability of the composite electrodes. As shown in Fig. 4c, the BC-G-TiO_2_ had the highest stability. The high electrochemical stability of TiO_2_ and its composites has been demonstrated, and the details have been discussed in our previous studies^44^. The lower stability of BC-G and BC may mainly be ascribed to the BC. As a three-dimensional carbon scaffold directly obtained using a carbonization process, the BC had relatively poor mechanical properties. Thus, an inevitable collapse and cracking occurred during the electrochemical process, which decreased the electrochemical properties^45,46^.

Many factors affect the rate performance of electrodes, including the structure, conductivity, and components of the electrode materials^47,48^. Figure 4d shows the good rate performance of the BC-G-TiO_2_. This can be ascribed to its unique structure, which included (i) the structure of the TiO_2_ nanoparticles distributed on the surface of the millimeter-scale porous BC scaffold. Hu *et al*.^49^ indicated that the diffusion distance of the electrolyte to a pseudocapacitor electrode surface was approximately 20 nm. Thus, this millimeter-scale structure contributed an abundance of accessible electroactive sites for the electrolyte. Meanwhile, TiO_2_ nanoparticles were encapsulated and connected by graphene and distributed on the surface of the BC, this structure facilitated the electron transfer within the BC-G-TiO_2_, improved the efficiency of the redox reaction of TiO_2_, and maintained the high electrochemical activity of the TiO_2_. (ii) the graphene and carbonized loofah fiber provided an efficient ion-transport pathway. Thus, the multi-functionalities of the BC-G-TiO_2_ composite delivered a high rate performance.

In summary, the introduction of this BC scaffold directly derived from a loofah as the three-dimensional skeleton of BC-G-TiO_2_, realizes the facile ions-transport all through the entire porous structure and the increase of ion-accessible surface area. This scaffold not only played a significant role in the high electrochemical performance of the BC-G-TiO_2_, but also provided an eco-friendly, novel and promising network scaffold with potential use as an electrode for a supercapacitor.

## Conclusions

A completely natural highly interconnected and interpenetrated loofah-derived hierarchical scaffold for making three-dimensional BC-G-TiO_2_ composite electrodes for supercapacitors was succeeded. The composite electrode exhibited good electrochemical performance of 250.8 F g^−1^ at a current density of 2 A g^−1^. The BC scaffold facilitated the electron transfer and increased the ion-accessible surface area for the electrolyte, providing a high EDLC and good rate performance. TiO_2_ remarkably improved the pseudocapacitance and electrochemical stability of BC-G-TiO_2_. The graphene improved the conductivity and capacitance of the composite electrode. The introduction of this novel three-dimensional scaffold derived from a loofah will broaden the scope of research on related materials, and provide inspiration for the design of composite electrode materials for supercapacitors.

## Experimental Methods

### Synthesis of graphene oxide (GO)

GO was synthesized using a modified Hummer’s method. The details have been reported in our previous literature^50,51^.

### Treatment for loofah precursor

The skin of a matured loofah was peeled off and soaked in deionized (DI) water to remove the loofah organisms until the inner biological loofah fiber network was obtained. Then, the as-prepared loofah fiber network was washed several times with DI water and alcohol, and then dried.

### Preparation of Titania precursor nanoparticles

Titania precursor nanoparticles were fabricated using a sol-gel method. The details were reported in our previous papers^44,52^. Briefly, the titania precursor solution was prepared by dissolving tetrabutyl [Ti(OC_4_H_9_)_4_] in an ethanol solution, and then adding hydrochloric acid as a solution stabilizer. Next, the Ti(OC_4_H_9_)_4_ was hydrolyzed and polycondensed to form a TiO_2_ gel. The reactions are shown in the following equations:

2 $$Ti{(OR)}_{4}+X{H}_{2}O\to Ti{(OH)}_{y}{(OR)}_{4-y}+zROH$$

3 $$Ti-OH+Ti-OR\to -Ti-O-Ti-+ROH$$

4 $$Ti-OH+Ti-OH\to -Ti-O-Ti-+{H}_{2}O$$

where R stands for C_4_H_9_. The gel was dried overnight and ground to obtain titania precursor nanoparticles (Ti_x_O_y_).

### Preparation of bio-carbon-based composite

As shown in Fig. 1, a simple process was utilized in this study to acquire the carbon-graphene-TiO_2_ composite. First, 0.15 g of Ti_x_O_y_ was mixed in 20 ml of a GO solution (5 mg ml^−1^). Second, the loofah precursor was cut into small pieces (1 × 1 × 5 cm^3^), and one piece of loofah precursor was immersed in the prepared solution. The loofah precursor was squeezed and loosened to absorb the mixed solution onto its fiber network structure just like a sponge. After being squeezed and loosened several times, the loofah was dried at 70 °C. This process was repeated until all of the GO and Ti_x_O_y_ mixed solution was absorbed to acquire the loofah-GO-Ti_x_O_y_ composite. Finally, a high-temperature treatment was carried out at 900 °C in an argon atmosphere for 2 h to implement the carbonization of the loofah, reduction of the GO, and crystallization of the TiO_2_ and finally obtain a composite with bio-carbon fiber network, reduced graphene, and TiO_2_, which was labeled BC-G-TiO_2_. The same process was also applied in the preparation of a control group containing a pure carbonized loofah fiber network labeled BC, and carbonized loofah fiber network that incorporated reduced graphene oxide, labeled BC-G.

### Characterization methods

A scanning electron microscope (SEM; INSPECT-F, FEI, The Netherlands) equipped with an energy dispersive X-ray spectroscopy (EDX) (Oxford Instrument, UK) was used to analyze the morphology of the BC-G-TiO_2_ composite electrode. Trans-mission electron microscopy (TEM, Tecnai G2 F20 S-TWIN) with an acceleration voltage of 200 kV was used to characterize the morphology of samples. The crystalline phase of the as-prepared TiO_2_ was identified using an X-ray diffractometer (XRD; X′pert pro-MPD, PANalytical, The Netherlands). The XRD measurements were performed on a stage using a Cu-Kα (wavelength, 1.5056 Å) X-ray source with a step rate of 0.02° s^−1^. The total pore surface area of each three-dimensional composite was measured by a mercury intrusion porosimetry method, which was conducted using an Autopore IV 9500. The volume of mercury was accurate to 0.1 µL.

An electrochemical analytical system (IM6, Zahner elektrik GmbH, Germany) was used for the measurements of electrochemical performance of all the samples. The cyclic voltammetry (CV) was conducted within the potential range from 0 to 1 V in electrolyte of 1 M H_2_SO_4_ at room temperature. The scan rate of 5, 10, 20, and 40 mV s^−1^ was used during the CV process. The galvanostatic charge-discharge measurement was conducted in the potential range of 0 to 1 V in the same electrolyte as CV. The gravimetric specific capacitance ${C}_{sp}$ (F g^−1^) of all the samples was calculated from each galvanostatic charge-discharge curve according to Equation (5):

5 $${C}_{sp}=\frac{It}{m{\rm{\Delta }}V}$$

where *I* is the constant current, *t* the discharged time, *m* the mass of each as-prepared sample, and ${\rm{\Delta }}V$ the width of the voltage window^53^.

## Electronic supplementary material

Supplementary Information
