# Supplementary material for: Bio-derived three-dimensional hierarchical carbon-graphene-TiO2 as electrode for supercapacitors
Source: Sci Rep. 2018 Mar 13;8:4412. doi: 10.1038/s41598-018-22742-7 (PMC5849706; doi:10.1038/s41598-018-22742-7)
Supplement: Supplementary file 1 — Supplementary Information [file 41598_2018_22742_MOESM1_ESM.pdf]

Bio-derived three-dimensional hierarchical carbon-graphene-TiO_2 as electrode for supercapacitors

Lili Jiang[1], Zhifeng Ren*[,2] Shuo Chen[2], Qinyong Zhang[1], Xiong Lu[3], Hongping Zhang[4], and Guojiang Wan[3]

1.Key Laboratory of Fluid and Power Machinery of Ministry of Education, Center for Advanced Materials and Energy, School of Materials Science and Engineering, Xihua University, Chengdu 610039, China

2.Department of Physics and TcSUH, University of Houston, 3201 Cullen Blvd Houston, Texas 77204, United states.

3. Key Lab of Advanced Technologies of Materials, Ministry of Education, School of Materials Science and Engineering, Southwest Jiaotong University, Chengdu 610031, Sichuan China

4.School of Materials Science and Engineering, Southwest University of Science and Technology, Mianyang 621010, Sichuan China

* Corresponding author, E-mail address: zren2@Central.UH.EDU

Support information

Figure S1 shows the EDX mapping image of BC-G-TiO_2. Figure S1a is TEM image of BC-G-TiO_2, while Figure S1b indicated the EDX mapping image of all C, Ti, and O elements. The distribution of C, Ti, and O are shown in Figure S1c, d, and e. These results prove that the TiO_2 nanoparticles are evenly distributed.

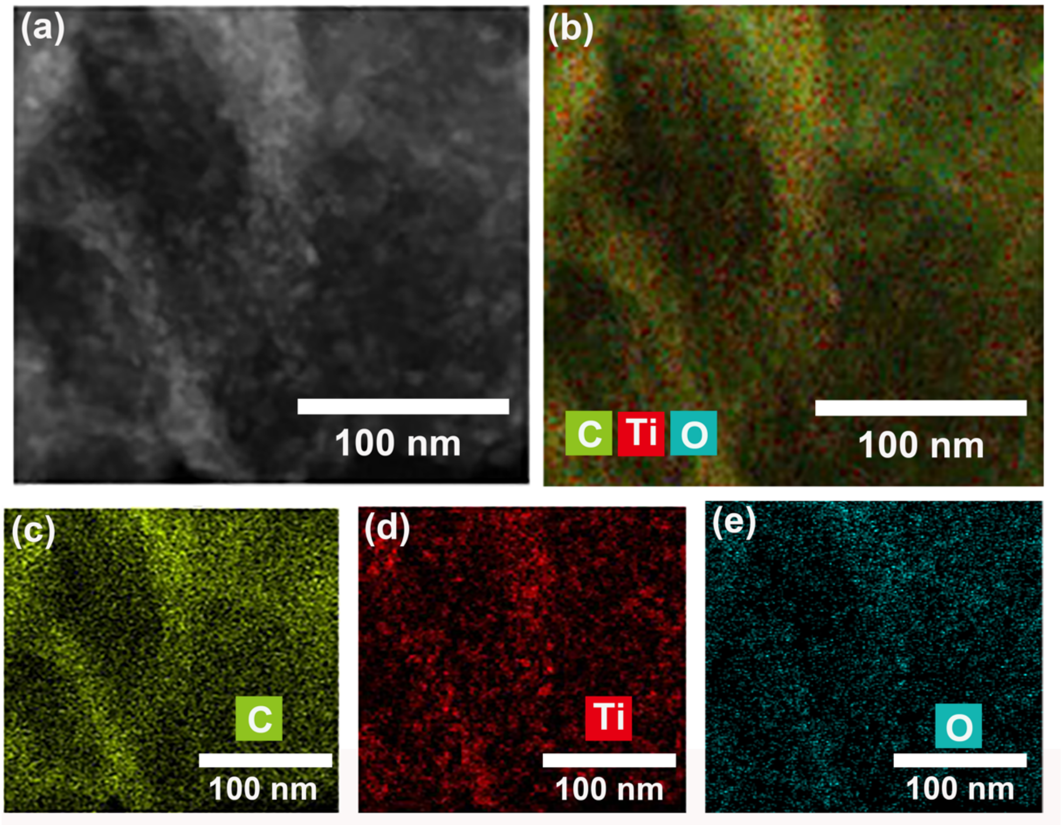

Figure S1 (a) TEM images of BC-G-TiO_2; (b) EDX mapping of BC-G-TiO_2; (c), (d), and (e) EDX mapping of the distribution of elements C, Ti, and O.
